# Leveraging the translational science benefits model to enhance planning and evaluation of impact in CTSA hub-supported research

**DOI:** 10.3389/fpubh.2025.1593920

**Published:** 2025-06-05

**Authors:** Andrea Molzhon, Pamela M. Dillon, Deborah DiazGranados

**Affiliations:** ^1^C. Kenneth and Dianne Wright Center for Clinical and Translational Research, Office of the Vice President for Research and Innovation, Virginia Commonwealth University, Richmond, VA, United States; ^2^Department of Psychiatry, Virginia Commonwealth University, Richmond, VA, United States

**Keywords:** research impact, evaluation, translational science, CTSA, impact evaluation models, translational science benefits model

## Abstract

**Introduction:**

Increasingly, the public, policymakers, and funders expect clinical research to show tangible effects on public health. However, assessing research impact is challenging. Most researchers are not trained to consider the broad-ranging impacts of their work. The TSBM is a conceptual framework that includes four domains of impact: clinical, community, economic, and policy. We assess the utility and acceptability of using a survey based on the TSBM as a means to help researchers identify their potential research impacts.

**Methods:**

CTSA program-supported investigators self-reported the potential benefits of their research projects in an electronic survey based on the TSBM. Responses were reviewed and scored by program evaluators. Survey acceptability was measured by response and completion rates; utility was measured by comparing benefits identified in the survey but not described in the researcher’s grant application; and quality was measured by the degree of congruence between investigators’ responses and evaluators’ determinations regarding the potential benefits of the research.

**Results:**

Of the investigators invited to participate, 67% completed the survey. Half of the investigators identified at least one benefit from their research not described in their research proposals. The rate of agreement across all responses between the investigators and the evaluators was 60%.

**Discussion:**

Our study showed that a survey based on the framework of the TSBM was an acceptable and useful tool to help investigators identify research impact. However, our work also suggested that there are opportunities to educate investigators especially about the long-term, broad-reaching effects of their work. Ultimately, this work may help researchers conceptualize and realize the public health impact of their research.

## Introduction

In the past decade, there has been an increasing call to evaluate the impact of research and to equip researchers with the skills necessary to enhance the reach and influence of their work (1, 2). As competition for research funding grows, it is essential to demonstrate that research contributes meaningfully to society and the broader world. However, assessing research impact is inherently complex—both in defining what constitutes impact and in tracking its wide-ranging effects. Most discussions of research impact focus on its benefits (3), yet capturing these effects requires establishing clear links between research outputs and tangible outcomes. While research may not be the sole driver of a given impact, it must be shown to be a necessary component of change (2, 4).

Impact evaluation involves identifying both intermediate and downstream effects of research, often requiring multiple forms of evidence (2, 5). The assessment of impact considers both its significance, the magnitude of the effect and its reach, and the size and composition of the populations affected. One approach to evaluating research impact is through indicator-based methods, which use measurable outputs to assess the extent to which research has contributed to observed outcomes. If research outcomes align with anticipated effects, this can strengthen claims of causality or impact. In this paper, we apply an indicator-based approach based on a published framework, the Translational Science Benefits Model (TSBM; 6), to evaluate investigators’ conceptualizations of the impact of the research produced by a Clinical and Translational Science Award (CTSA) program. We report on the utility of applying the selected framework to helping investigators anticipate and define various potential benefits of their research and to assess investigators’ abilities to interpret the expected results of their research.

Clinical and translational science (CTS) aims to bridge the gap between research and practice, transforming scientific discoveries into improved clinical practice, policies, and health outcomes (6). While the short-term, academic impacts of research can be quantified through publications, citations, and subsequent research funding, measuring the broader, downstream effects of research on human health remains challenging (7–9). Unlike academic outcomes, research impact, which encompasses patient, community, and societal benefits resulting from research, is harder to link directly to a single project or even a researcher’s body of work. The reasons for this are many-fold and include the complexity of factors impacting health, the temporal distance between research and clinical implementation, and the rapidly changing clinical, regulatory, and policy environment (2). Despite these challenges, an assessment of research impact is critical to fully understand the value of research to public health.

**Figure 1:**
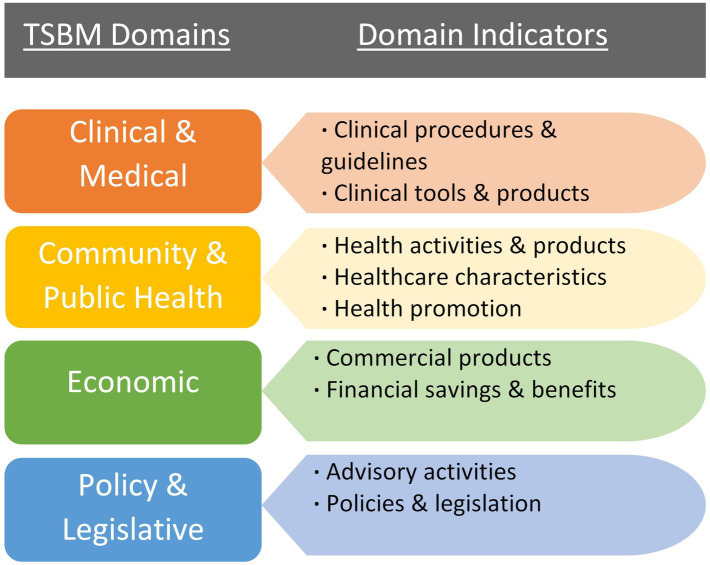
The domains and components of the TSBM (11).

Researchers often lack training in capturing the broader, long-term impacts of their research, yet funding agencies increasingly require grant applications to articulate potential research impacts (10). This highlights the need for tools and frameworks that enable researchers to better understand and measure the full spectrum of their work’s impact, such as how research informs or influences clinical care guidelines, health policies, and community health initiatives. The TSBM (11) offers a conceptual framework for this purpose. The TSBM is a multifaceted approach to defining the benefits that could result from CTS research. As illustrated in Figure 1, the model includes four broad domains of impact/benefit with indicators of specific impacts/benefits for each domain. This provides a clear framework for researchers to plan for, assess, and track ways their research may be used to benefit a broad range of stakeholders, such as practitioners, community organizations, and policy makers. Evidence suggests that the model may be useful particularly for researchers who are in the early stages of the research process (12). Though, while there is clear evidence that the model can be used effectively as a framework for the presentation of case studies (12–14), evidence that the model can be used as a tool to improve researchers’ competencies in impact measurement is limited (12). Despite the potential added value of assessing impact beyond academic publications and grants, the TSBM is a relatively new evaluative framework in CTS, so it is uncommon for researchers to receive targeted guidance or training to use the model for research planning and evaluation.

The project is being conducted under a CTSA, a seven-year program grant awarded by the National Institutes of Health’s (NIH) National Center for Advancing Translational Sciences (NCATS) to support and advance CTS. This paper describes the initial phase of an ongoing project to improve our evaluation of the public health impact of the work of our CTSA program. The broad goal of this paper is to examine and describe our CTSA program-supported researchers’ ability to conceptualize the potential impacts of their CTS research. The specific objective of this phase of our impact evaluation project is to evaluate the utility and acceptability of collecting self-reported research impact data using a survey based on the TSBM framework. We surveyed CTSA-supported investigators who were beginning their research projects, as well as those who had recently completed their CTSA-supported projects. The findings will be used to help our CTSA program develop new resources and training opportunities for researchers to improve how they consider, plan for, and track a broad array of near- and long-term benefits resulting from their research, ultimately optimizing the public health impact of their work. The findings also will form a basis for the development of a robust framework to evaluate the research impact of our CTSA-funded program.

## Methods

### Design and participants

We conducted a cross-sectional descriptive study in which investigators supported by our CTSA hub who were awarded pilot funding or training grants (e.g., K scholars or Supplement Scholar Awards) were recruited to participate in an electronic survey based on the TSBM. We began collecting survey data in May 2024.

### Measures

We utilized a survey that was informed by the TSBM framework. The survey was based on an instrument developed at Case Western University and adapted to align with our specific evaluation needs (15). Surveys were electronically completed using Research Electronic Data Capture (REDCap; 16), a secure, web-based survey platform. The survey was designed to be an investigator’s self-assessment of their project’s potential impacts across the domains of the TSBM. First, items were included to capture information on the respondent’s project (i.e., project dates, title, aims, etc.); this data provided context about the project for the evaluators. Second, respondents were directed to, “Use this checklist to identify the anticipated/potential CLINICAL (including TOOLS & PRODUCTS), COMMUNITY, ECONOMIC, and/or POLICY benefits of your work,” and the checklist included nine yes/no items that aligned with each indicator of the four domains of the TSBM, as depicted in Figure 1: (1) Clinical and Medical (one indicator/checkbox), (2) Clinical and Medical Tools and Products (one indicator/checkbox), (3) Community and Public Health (three indicators/checkboxes, combined to form one score), (4) Policy and Legislation (two indicators/checkboxes, combined to form one score), and (5) Economic (two indicators/checkboxes, combined to form one score). We separated the domain of *clinical and medical benefits* into two parts to increase the accuracy and specificity of the data we collected (i.e., *clinical and medical*, and *clinical and medical tools and products*). For each potential benefit endorsed with a *yes* response, an open-text field appeared and respondents were asked to provide a description of those potential benefits.

### Data collection procedure

Surveys were electronically sent to our CTSA program-supported investigators (e.g., K12 Scholars, Diversity Supplement Scholars, and TS Pilot Awardees) via email followed by up to five reminders to investigators who did not initially respond.

### Survey analysis

We analyzed survey responses to identify and summarize indicators of survey acceptability, utility/value, and response quality. Evaluating acceptability, utility, and response quality was essential because the survey serves as a primary measure of impact within our evaluation plan and provides key information that will be used to inform training for investigators on research impact. Survey acceptability and utility/value were scored by the first author (AM). For response quality, we used a score-rescore method in which two evaluators scored investigator responses (AM and PD) in 100% of completed surveys. When scores differed between evaluators, scores were discussed until a consensus was reached.

#### Survey acceptability

Survey acceptability was defined as the willingness of investigators to take part in the TSBM survey. Understanding how willing investigators were to participate in the survey allowed us to assess its feasibility as a data collection tool. We calculated acceptability as response rates (percent of those who responded to the survey compared to those who were invited to participate) and completion rates (the percent of submitted complete surveys).

#### Survey utility/value

Survey utility was defined as the value or usefulness of the survey in helping respondents identify potential benefits of their research that they previously had not identified in their research applications. To measure this, we compared responses on the *potential benefits* survey items with the language included in the respondents’ research award applications. Each yes/no item that was endorsed with a *yes* response and was previously described in the grant application or their abstract received a score of *0*, as this indicated that the TSBM survey did not serve as a mechanism for investigators to conceptualize and describe the potential benefits of their research beyond what the investigator previously had articulated. Each benefit domain that was endorsed in the survey but not articulated in the corresponding award application received a score of *1*. As there were nine yes/no *potential benefits* indicators, survey utility scores ranged from 0 (low utility) to 9 (high utility).

#### Survey response quality

Response quality was defined as the perceived accuracy of investigator-endorsed or -unendorsed benefits across each TSBM domain. To measure response quality, we compared responses on each of the five *potential benefits* sections of the survey (i.e., Clinical and Medical, Clinical and Medical Tools and Products, Community and Public Health, Policy and Legislation, and Economic) with the language in the corresponding research award applications. If the evaluators identified a potential benefit of the investigator’s research that was not endorsed by the investigator in a given section, the score for that section would be 0. If the evaluators agreed with the investigator’s response, the score for that section would be 1. Response quality scores for each survey were calculated by counting the number of times the evaluators’ determinations matched the investigators’ responses across all survey sections. As there were five sections, *response quality* scores ranged from 0 (poor quality) to 5 (excellent quality).

## Results

### Participants

Seven investigators whose research projects were in the beginning stages (78% response rate) and three investigators whose projects had recently concluded completed a survey (50% response rate). See Table 1 for a summary of the groups of principal investigators (PIs) supported by our CTSA program who received and completed the survey.

**Table 1:** Survey distribution and completion counts by survey type and investigator group.

Investigator group	Distributed (*n*)	Completed (*n*)
2024–2025 and 2023–2024 pilot awardees	7	5
2025–2027 and 2022–2024 K12 scholars	6	3
2024–2025 diversity supplement scholars	2	2

### Survey acceptability

Of the 15 invited to participate, 11 responded (73.3%, response rate) and 10 (66.7%) completed the survey. This indicates that most investigators were willing and able to dedicate time to the survey.

### Survey utility/value

In 5 (50%) of completed surveys, there was at least 1 instance in which investigators identified a potential or demonstrated benefit of their research that they had not described in their grant application. This occurred at least once for every type of benefit domain represented in the TSBM. For example, one survey respondent expanded on what was included in their award application in the *clinical and medical* benefits domain, mentioning not only the potential benefits to *therapeutic procedures* (previously mentioned in award application), but also described potential benefits to *investigative procedures* that had not been described previously in their award application. Of the 5 survey respondents who endorsed at least one new potential benefit of their research, utility scores ranged from 1 to 4 (maximum score of 9) with an average score of 2.2. This indicates that, in 50% of survey respondents, the survey helped them to identify an average of 2 benefits that they previously had not articulated in their funding applications.

### Survey response quality

Across all completed surveys, investigators endorsed *clinical and medical* benefits most frequently (endorsed in 100% of completed surveys) and endorsed *policy and legislative* benefits the least frequently (endorsed in 20% of completed surveys); see Table 2. In 100% of the surveys, evaluators disagreed with at least one determination that the investigators made. The overall rate of agreement between the evaluators and the investigators across all responses was 60%. The evaluators determined that the investigators missed a potential benefit of their study that was identified by the evaluators in 30% of responses. In 6% of responses, the evaluators determined that benefit(s) endorsed by the investigators were incongruent with what one would expect from the described research project. In 4% of responses, the evaluators agreed with investigator endorsements but did not agree with the justification that the investigator provided. The *community and public health* benefits domain comprised the highest percentage of missed endorsements or incongruent responses from investigators (40%), followed by *economic* benefits (30%), and the *clinical and medical* benefits domain comprised the fewest number of missed endorsements or incongruent responses (5%). Of the items in the *community and public health* benefits domain that were flagged by evaluators, 75% were due to benefits identified by the evaluators but not the investigator (missed endorsements). Missed endorsements accounted for 100% of evaluator-flagged responses in the *economic* benefits domain.

**Table 2:** Investigators’ endorsements of benefits across TSBM domains.

	Benefit domain				
	Clinical and medical	Tools and products	Community and public health	Economic	Policy and legislative
Count of investigator endorsements					
Potential benefits (respondents = 10)	10 (100%)	5 (50%)	4 (40%)	3 (30%)	2 (20%)
Incongruent investigator endorsements					
Count (% of investigator endorsements)	1 (1%)	0	1 (25%)	0	1 (50%)
Missed endorsements by investigators					
Count (% of surveys)	0	2 (20%)	6 (60%)	6 (60%)	1 (10%)

## Discussion

Research impact is increasingly recognized as a critical component of scholarly work, shaping funding decisions, policy developments, and societal advancements. As research funders and governments worldwide demand evidence of public benefits from research investments, impact assessment has become essential for demonstrating the value of academic work (2). Evaluating research impact is important for ensuring accountability, optimizing resource allocation, improving research translation into practice, and fostering interdisciplinary collaboration. However, measuring research impact remains complex due to its subjective nature, diverse beneficiaries, and the challenges of attribution. Current methodologies for impact evaluation vary widely, from experimental and statistical methods to qualitative, systems-based, and indicator-driven approaches, each offering different insights into the significance and reach of research contributions. Despite the growing emphasis on impact evaluation, many existing frameworks struggle to capture the full spectrum of research benefits, particularly in fields where impacts are indirect, long-term, or difficult to quantify. Moreover, there is limited evidence on how to effectively train investigators to consider, plan for, and track a broad array of near- and long-term benefits resulting from their research. Addressing this gap is essential for optimizing the public health impact of research by equipping investigators with the tools to systematically integrate impact considerations into their work.

In this study, we used a survey based on the TSBM framework to capture self-reported descriptions of potential impacts. Responses were analyzed to identify and describe our CTSA program-supported researchers’ abilities to conceptualize the potential impacts of their CTS research across the four domains of the TSBM. This study is the first to our knowledge to assess investigator understanding of research impact using the TSBM framework. We found that completing the TSBM survey enabled investigators to identify more potential benefits of their research than they initially had articulated in their research applications. This speaks to the potential advantages of providing investigators with a guide to help them conceptualize and define the potential impacts of their work. Encouraging investigators to consider a more expanded view of the potential benefits of their research could influence their dissemination practices, perhaps helping to close the gap between translating research into practice. Our results add to the current literature on impact evaluation by contributing to the understanding of investigators’ perceptions of impact and providing insights into how to effectively frame the evaluation of research impact to capture both near- and long-term benefits.

## Limitations

The sampling technique used in this study limited the results in several ways. As our sample was small in size and non-randomized, selection bias may affect the wider generalizability of the findings and implications. Our sample consisted of CTS researchers supported by a university-based CTSA program who voluntarily completed the survey, so the findings may not generalize to all types of researchers or investigators in varied settings and under varied conditions. However, this was an exploratory, quality improvement study meant to inform the development of new resources and initiatives specifically for the CTS researchers at our CTSA program hub, so the findings were not intended to generalize to the wider population. Moreover, due to our small sample size, we were unable to examine possible moderating effects of individual-level variables such as award type, career stage, research experience, professional position, or other demographic-level variables. Future efforts to examine investigators’ conceptualizations of research impact within the context of the domains of the TSBM could include investigators from multiple CTSA programs to generate a larger, more representative sample that would allow for the examination of potential moderating factors.

The survey measure, itself, and the procedure used to score the survey also contributed to the study’s limitations. The survey primarily is meant to be used as a tool for self-reflection and self-report rather than for evaluation, therefore, scoring was subjective. Consensus-scoring served to minimize the subjectivity of the scores that were assigned. As the survey requested project information from the respondent and the two scorers were well-acquainted with the pilot projects of each survey respondent by virtue of their roles in the CTSA program (e.g., CTS pilot program director and evaluator) blinding was not possible. However, the background knowledge that the two scorers had in relation to each investigator’s project meant that scoring decisions were informed by a clear understanding of the research, and the two-scorer consensus process mitigated potential biases in scoring.

### Future directions

We already have begun the next phase of this research, which involves conducting investigator interviews. These interviews will gather feedback on investigators’ impressions of the TSBM-based survey, their current methods of tracking their research impact, and their ideas for how our CTSA hub can better support impact evaluation using the TSBM as a guiding framework. The broad goal of this research is to improve impact evaluation in our CTSA program. The specific goals of the second phase are to refine the survey to improve its clarity and utility and to develop new resources and/or training opportunities to assist investigators in understanding, measuring, and describing their research impact. Ultimately, this effort seeks to help investigators conceptualize and realize the multifaceted impacts of their research on public health.

## Conclusion

Researchers face challenges in identifying, tracking, and articulating the broader significance of their work. This study provides an initial understanding of how to better inform and support investigators in this process. By capturing investigators’ perceptions of research impact, our findings lay the groundwork for developing targeted resources and training opportunities.

## Data Availability

The datasets presented in this article are not readily available because the Virginia Commonwealth University Institutional Review Board (IRB) designated this project as “not human subjects research” due to it being a quality improvement project. Requests to access the datasets should be directed to diazgranados@vcu.edu.
